# Unsupervised Segmentation of Knee Bone Marrow Edema-like Lesions Using Conditional Generative Models

**DOI:** 10.3390/bioengineering11060526

**Published:** 2024-05-22

**Authors:** Andrew Seohwan Yu, Mingrui Yang, Richard Lartey, William Holden, Ahmet Hakan Ok, Sameed Khan, Jeehun Kim, Carl Winalski, Naveen Subhas, Vipin Chaudhary, Xiaojuan Li

**Affiliations:** 1 Program of Advanced Musculoskeletal Imaging (PAMI), Cleveland Clinic, Cleveland, OH 44195, USA; yangm@ccf.org (M.Y.); larteyr@ccf.org (R.L.); holdenw2@ccf.org (W.H.); oka4@ccf.org (A.H.O.); khans24@ccf.org (S.K.); kimj14@ccf.org (J.K.); winalsc@ccf.org (C.W.); subhasn@ccf.org (N.S.); lix6@ccf.org (X.L.); 2 Department of Biomedical Engineering, Lerner Research Institute, Cleveland Clinic, Cleveland, OH 44195, USA; 3 Department of Computer and Data Sciences, Case Western Reserve University, Cleveland, OH 44106, USA; vxc204@case.edu; 4 Department of Electrical, Computer, and Systems Engineering, Case Western Reserve University, Cleveland, OH 44106, USA; 5 Department of Diagnostic Radiology, Imaging Institute, Cleveland Clinic, Cleveland, OH 44195, USA

**Keywords:** unsupervised segmentation, anomaly detection, deep learning, generative adversarial networks, diffusion models bone marrow edema-like lesions, MRI, inter-rater reliability

## Abstract

Bone marrow edema-like lesions (BMEL) in the knee have been linked to the symptoms and progression of osteoarthritis (OA), a highly prevalent disease with profound public health implications. Manual and semi-automatic segmentations of BMELs in magnetic resonance images (MRI) have been used to quantify the significance of BMELs. However, their utilization is hampered by the labor-intensive and time-consuming nature of the process as well as by annotator bias, especially since BMELs exhibit various sizes and irregular shapes with diffuse signal that lead to poor intra- and inter-rater reliability. In this study, we propose a novel unsupervised method for fully automated segmentation of BMELs that leverages conditional diffusion models, multiple MRI sequences that have different contrast of BMELs, and anomaly detection that do not rely on costly and error-prone annotations. We also analyze BMEL segmentation annotations from multiple experts, reporting intra-/inter-rater variability and setting better benchmarks for BMEL segmentation performance.

## 1. Introduction

Osteoarthritis (OA) is the most common form of arthritis and a leading cause of diminished quality of life and disability, affecting over 32 million U.S. adults. In 2013, the total economic burden of OA, including medical interventions, mood disturbances, and work limitations, was estimated to be more than $373 billion annually in the U.S. alone [1]. The knee is the joint most frequently affected by OA, particularly among the elderly. However, the global prevalence of knee osteoarthritis (KOA) in people aged 15 and above is still high at 16.0%, caused by a wide variety of risk factors including age, gender, obesity, genetics, diet, abnormal loading, and malalignment [2]. With the global population growing older and the KOA incidence rate rising beyond 203 per 10,000 person-years for people 15 and above, the impact of KOA is likely to become more significant in the future [3].

Currently, there is no effective cure for KOA. However, it may be manageable and preventable if its early signs are detected before the symptoms become debilitating or disabling [4]. In working towards an effective treatment, reliable and sensitive methods for early diagnosis and prognosis of KOA are essential. High resolution medical imaging, particularly magnetic resonance imaging (MRI), offers a powerful tool for such methods since it provides information regarding the state of a knee joint before the disease has progressed significantly and symptoms are detectable externally, such as with physical tests and patient-reported pain assessment scales. Other non-invasive diagnostic methods such as X-ray [5] and vibroarthrography [6,7,8], have been explored as well, though X-ray has low sensitivity when detecting KOA in the critical early stages [9] and vibroarthography is still being explored [10].

MRI have been used extensively to study KOA, which provide evaluation of multiple tissues within the joint, including cartilage, meniscus, bone, ligament, tendon, muscle, synovium and fat [11]. Bone marrow edema-like lesions (BMEL) are of particular interest in this paper. BMEL refers to regions of high-intensity edema/fluid-like signals in fluid-sensitive MRI (normally T2-weighted fat-suppressed spin-echo MRI), surrounded by healthy and low-intensity marrow which consists of primarily fatty tissues. Although termed as *edema*, previous histological studies showed that these lesions have other pathological features including abnormal trabeculae, bone marrow necrosis, swelling of fat cells, and marrow bleeding [12].

BMEL presence in the knee joint has been associated with KOA progression and pain [13,14], including after acute injuries such as anterior cruciate ligament and meniscus tear injuries that lead to post-traumatic OA [15,16,17]. Evaluations of BMEL in the literature have been dominated by semi-quantitative grading by radiologists, using scoring systems such as WORMS and MOAKS [18,19]. However, readings from expert radiologists are expensive, time-consuming, and prone to intra-/inter-rater variability. Furthermore, semi-quantitative grading has limited sensitivity of detecting small but significant longitudinal changes. Fully quantitative evaluations like volume and signal intensity of BMEL are desired [13], which first requires segmentation. Manual and semi-automatic methods have been developed to segment BMEL [20], but their utilization has been impeded by the labor-intensive and time-consuming nature, as well as by annotation bias and intra-/inter-rater variability. More recently, efforts have been undertaken to develop automatic segmentation methods for BMELs [21,22,23]. However, they suffer from limited training data, pre- and post-processing requirements, and annotation requirements. These challenges are due primarily to the nature of BMELs as they are visualized by MRI:

- Small size relative to the MRI field-of-view
- Large variance in size and shape across different instances
- Diffuse intensity levels and unclear boundaries
- Disagreement among experts on their presence and boundaries

**Figure 1:**
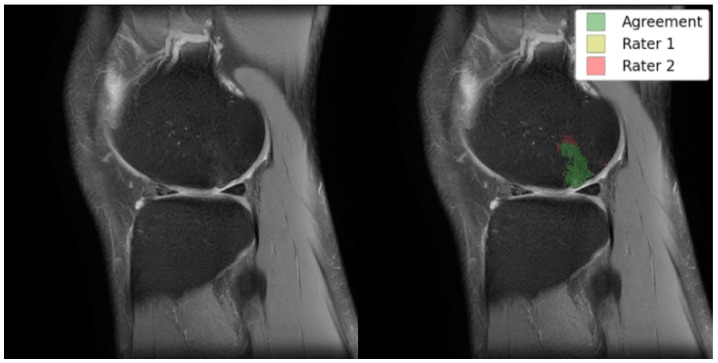
Left: original TSE image; right: TSE image with an overlay of BMEL annotations by two raters; Rater 2 marked the anterosuperior boundaries of the BMEL to extend further than Rater 1. TSE: turbo-spin echo.

As exemplified in Figure 1, these characteristics of BMELs may result in their expert annotations having poor intra- and inter-rater reliability. The poor reliability of the annotations poses a significant risk to any deep learning models trained on such unreliable targets, because the trained model will carry the biases and errors of the annotations when they learn to reproduce the segmentation masks. This is a fundamental challenge to supervised deep learning tasks, sometimes summarized as garbage in, garbage out. To the best of the authors’ knowledge, no research has been done to address the poor intra- and inter-rater reliability of BMEL annotations, nor to identify or quantify their extent, in the context of segmentation models and quantitative analysis.

Unsupervised learning is a deep learning technique by which a model learns to make predictions on unseen data points without any labeled data. Instead, patterns in the data are extracted, and labels may be placed on the pattern clusters post-hoc. This study explores a specific form of unsupervised learning called anomaly detection, where a generative model learns the characteristics of healthy knee MRI, then determines whether an unseen image matches those healthy characteristics or not. A major advantage of unsupervised segmentation is its freedom from the costs, biases, and errors that are associated with manual annotations. It also benefits from the over-representation of healthy images that is commonly seen in medical image datasets. While a supervised learning model may not learn much from more samples in an already over-represented class, an unsupervised learning model can use them to further refine its understanding of the majority class, the healthy images.

The purpose of this study is to develop automated tools that use MRI for better diagnosis and prognosis of KOA. To that end, it introduces two fully automatic, unsupervised deep learning frameworks that demonstrate the feasibility of training and deploying an unbiased BMEL segmentation model. It also analyzes the intra- and inter-rater variability of BMEL segmentation annotations.

## 2. Materials and Methods

This section first details the subjects used in this study, including the methods and parameters used to acquire the MRI. Then, the image preprocessing steps are described, including registration and normalization. Steps taken by the expert annotators to create the segmentation annotations are listed, along with the definitions of quantitative metrics used to measure intra- and inter-rater reliability. Finally, the two deep learning frameworks are outlined, followed by details on the neural network architectures, training, validation, and testing schemes, and other configurations and hyperparameters used. Finally, the postprocessing steps used to generate the model predictions are also described.

### 2.1. Study Cohort and MRI Acquisition Protocol

177 patient knee MR images were sourced from two clinical cohorts: the Multicenter Orthopedics Outcomes Network (MOON) Onsite Cohort and the Corticosteroid Meniscectomy Trial of Extended-Release Triamcinolone after Arthroscopic Partial Meniscectomy (COMET), collected using two MRI vendor systems (Siemens and Philips) and four institutions (Cleveland Clinic, Brigham and Women’s Hospital, Vanderbilt University, and the Ohio State University). Sequences analyzed included fat-suppressed 2D turbo spin echo (TSE) and fat-suppressed 3D dual echo steady state (DESS) imaging. Imaging protocols were harmonized between sites and scanners using traveling volunteers and phantoms to ensure imaging reproducibility. Demographics data and sequence acquisition protocols for both cohorts are included in Table 1 and Table 2, respectively. Both the MOON and COMET studies received approval from the internal review board (IRB) at each participating institution, ensuring compliance with ethical standards for research involving human subjects.

### 2.2. MRI Preprocessing

To standardize the MR images across multiple clinical cohorts, sites, and vendors, several preprocessing steps are applied to the images. First, scaling is used to ensure each volume (all slices for each knee MRI) has an equivalent intensity value range, then clipped to the 0.001 and 0.999 quantiles to limit extreme intensity values, especially on the brighter end. This is done also to ensure the intensity values are well-distributed within the range [−1,1], matching the range of the hyperbolic tangent activation function in the final layer of the neural networks described in Section 2.5. Afterwards, the DESS images are registered to the TSE voxel space using Advanced Normalization Tools’ (ANTS) rigid registration algorithm [24] with a linear interpolator. Finally, the volumes are center-cropped from 320×320 to 256×256 voxels per slice along the sagittal plane. Due to the large field-of-view of the volumes, the cropping does not exclude any bone marrow regions or BMELs.

Another crucial preprocessing step is the generation of masks for the bone marrow region of each volume. Since BMEL only manifests inside the bone marrow, the bone masks can be applied in a variety of ways that will not affect the prediction of BMEL regions. This is akin to the generation and application of masks for the brain tissue in each brain volume, called skull-stripping. The bone masks in this study are generated by a separate, in-house 3D convolutional neural network based on UNet [25] with four down and up levels, each with batch normalization, ReLU activation functions, 3D max pooling, and dropout, configured with two conv-act-norm blocks and the dropout layer. The final activation function is softmax. This model was trained in a supervised manner using expert bone mask annotations on the DESS sequence, whose outputs are registered to TSE using the same rigid registration affine transforms that are computed for the corresponding DESS image.

**Figure 2:**
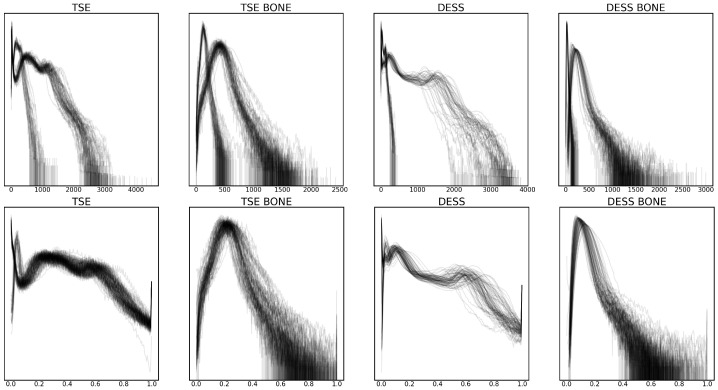
Smoothed intensity level histogram plots for each volume before (**top row**) and after (**bottom row**) preprocessing, with raw and normalized intensity values in the x-axis, respectively. The density of the histogram in the y-axis is log-scaled. Two sequences, turbo spin echo (TSE) and double echo steady state (DESS) are considered, as well as bone regions for each of the sequences (TSE BONE and DESS BONE).

These preprocessing steps were validated by plotting the intensity value histograms for each volume and bone region, as shown in Figure 2. Some of the notable changes are the standardization of intensity values in volumes with different bit depths after scaling, as well as the more evenly distributed intensity values after clipping.

### 2.3. BMEL Annotation and Evaluation

Though our proposed methods do not rely on expert annotations of BMELs for model training, the annotations are used to test the performance of the model. In addition, to test the intra-/inter-rater reliability of BMEL annotations, two trained research fellows produced annotations for the same set of testing volumes. In each participant, BMELs were identified as areas of high signal intensity on sagittal TSE images. This segmentation process was performed by two musculoskeletal imaging fellows under the training and supervision of an experienced musculoskeletal radiologist with over 30 years of expertise. The process started with manually outlining a rough region of interest around the BMEL to generate a mask. Subsequently, BMEL was automatically segmented within this region using a specific threshold, utilizing in-house software. Dice similarity coefficient (DICE) was used to quantify intra-/inter-rater reliability, given by Equation (1):

(1) $$\mathrm{DICE}=\frac{2\mathrm{TP}}{2\mathrm{TP}+\mathrm{FP}+\mathrm{FN}}$$

where TP, FP, and FN are true positive, false positive, and false negatives in binary classification, respectively. True negatives (TN) are not part of Equation (1) because it often skews the metric towards a perfect score when there is an overabundance in TNs. This issue well-described for other metrics such as accuracy, given by Equation (2):

(2) $$\mathrm{Accuracy}=\frac{\mathrm{TP}+\mathrm{TN}}{\mathrm{TP}+\mathrm{TN}+\mathrm{FP}+\mathrm{FN}}$$

### 2.4. Unsupervised Segmentation of BMEL

**Figure 3:**
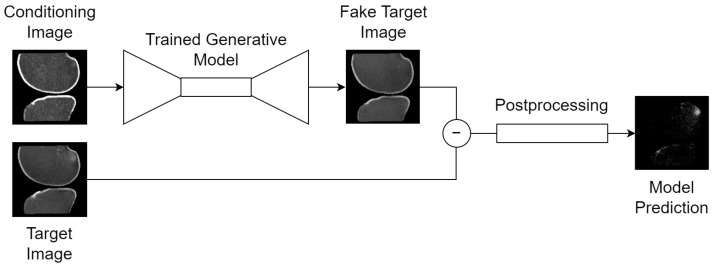
Generic inference scheme for all tasks: (1) a conditioning image is passed to the trained generative model, which guides its synthesis with information such as the orientation of the knee in voxel space; (2) the trained generative model synthesizes a fake, healthy version of the target image; (3) a voxel-wise subtraction of the real and fake target images generates an anomaly map; (4) deterministic postprocessing is applied to the anomaly map to produce the final model prediction: a binary segmentation map of the BMEL. Thumbnails exemplify the bone translation task outlined in Section 2.4.

The overall framework is outlined in Figure 3 and designed as follows: a conditional generative model is trained to synthesize healthy 2D slices of the knee MRI, conditioned on an image that imparts some information about the slice to synthesize. More generally, this task is called image-to-image translation since a conditioning image is passed to the model as input, and the model outputs another image. When the conditioning image is a different MRI sequence of the same slice, it is called sequence translation. When the conditioning image is the same MRI sequence of the same slice with some portions missing, it is called inpainting or uncropping.

**Figure 4:**
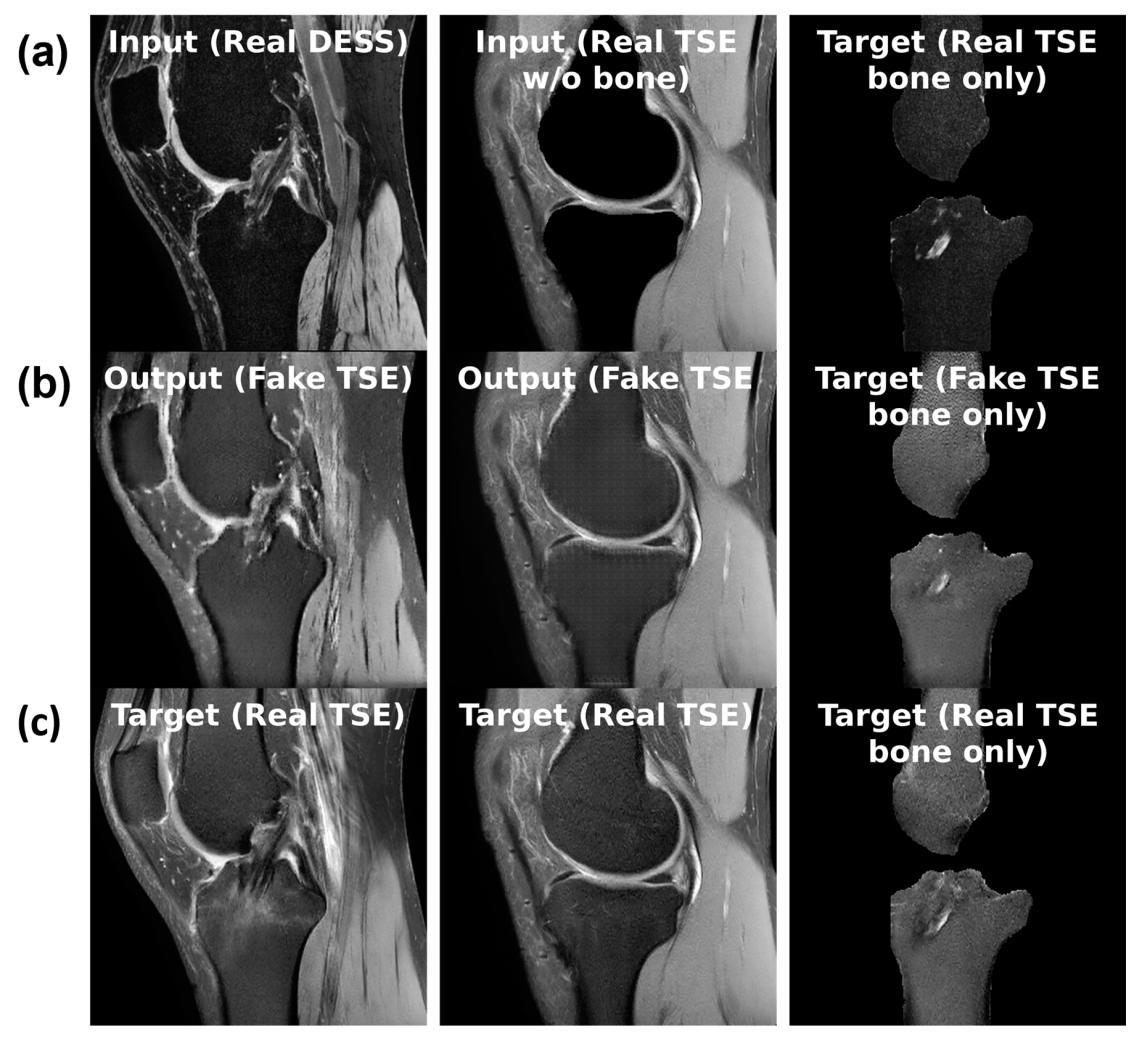
(**a**) Sample inputs (**top row**), (**b**) outputs (**middle row**), and (**c**) targets (**bottom row**) for each of the three tasks from left to right: slice translation; bone inpainting; and bone translation.

For the sequence translation task shown in Figure 4a, DESS is the input sequence and TSE is the output sequence. During training, the model gets a full view of the input DESS image and learns to synthesize the corresponding TSE image. Since these images are co-registered and most structures are consistent across the sequences, the model can learn this task. Furthermore, the training dataset only consists of healthy, BMEL-free images, which means the model will always synthesize a healthy TSE image given any DESS image, even if the real TSE image contains a BMEL. Therefore, DESS is the preferred input sequence since it has low signal intensity difference in healthy and BMEL regions; healthy and unhealthy DESS images look similar. Conversely, TSE is the preferred output sequence since it has high signal intensity difference in healthy and BMEL regions. The resulting anomaly map is computed by taking the difference between the real unhealthy TSE image and the fake healthy TSE image.

For the bone inpainting task shown in Figure 4b, both input and output sequences are TSE, except the input image has its bone regions removed using the bone segmentation masks. To reconstruct the full TSE image as output, the model learns to inpaint the bone given the surrounding regions, such as cartilage, muscle, etc. Again, the model is trained only on healthy images, which means it will inpaint a healthy bone region whether or not the real bone region contains BMEL or not. The bone mask is a suitable region to inpaint because by definition, BMEL occur inside the bone region and will always be inpainted.

Another task, called the bone translation task shown in Figure 4c, is also considered. In this case, only the bone regions of both DESS and TSE sequences are kept; non-bone regions are masked out using the bone segmentation mask. After this step, the task proceeds like sequence translation: DESS sequence is the input and TSE sequence is the output. This task may have the advantage of removing confounding factors in the surrounding regions around the bone that may not be relevant to the generation of the anomaly map.

During inference, the generative model is given an unseen slice. The model carries out its task to synthesize a healthy version of the input slice, regardless of whether the actual slice contains a BMEL or not. The voxel-wise difference between the synthesized and actual versions of this slice is its anomaly map, considered to be the raw output of this model. The anomaly map can then be postprocessed and binarized into a segmentation map.

From the perspective of the overall framework, the conditional generative model can be treated as a black box as long as it satisfies the above requirements for input and output images. This enables different classes of generative models to be dropped in and out of the framework with minimal adjustments. While keeping all other configurations the same, a conditional generative adversarial network (cGAN) and a conditional diffusion model (cDIFF) are trained on the three aforementioned tasks respectively, resulting in a total of six models. The performance of these six models are then evaluated and compared. More model details are given in Section 2.5 and Section 3.2.

### 2.5. Generative Model Classes

Two classes of conditional generative models, conditional generative adversarial networks (cGAN) and conditional diffusion models (cDIFF) were used in this study. Some details regarding their model design and implementation, as well their implications for this study, are given here.

#### 2.5.1. Conditional Generative Adversarial Networks

Generative adversarial networks (GAN), introduced by Goodfellow et al. in 2014 [26], represent a pivotal development in the field of unsupervised learning and generative models. Two neural networks are engaged in a zero-sum game inside each GAN, in which the generator *G* aims to synthesize data that is indistinguishable from real data, while the discriminator *D* tries to distinguish between real and synthesized data. Conditional GANs (cGAN) are an extension to GANs in which some additional conditioning data *c* from the real dataset is given to both *G* and *D* to constrain data synthesis and discrimination, respectively:

(3) $$x,c∼p_{data}\left(data\right);$$

(4) $$\min_{G}\max_{D}V^{\prime }\left(G,D\right)=E_{x,c∼p_{data}\left(data\right)}\left[\log D\left(x,c\right)\right]+E_{z∼p_{z}\left(z\right)}\left[\log\left(1-D\left(G\left(z,c\right)\right)\right)\right]$$

where $E_{x∼p_{data}\left(x\right)}\left[\log D\left(x\right)\right]$ is the expected value of the discriminator’s ability to identify real data, $E_{z∼p_{z}\left(z\right)}\left[\log\left(1-D\left(G\left(z\right)\right)\right)\right]$ is the expected value of the discriminator’s failure to identify synthesized data, and $z∼p_{z}\left(z\right)$ is a latent space vector drawn from a known prior distribution, usually Gaussian noise. *G* maps *z* to the data space: $G\left(z\right)=\hat{x}$ where $\hat{x}$ is synthesized data; *D* maps *x* to a single probability that *x* was drawn from the real dataset $p_{data}$ instead of the generator’s distribution $p_{g}$.

**Figure 5:**
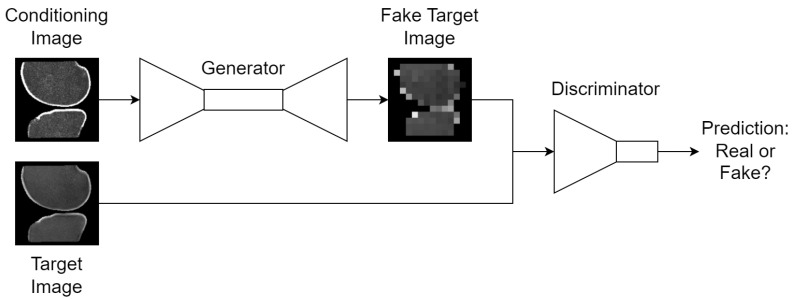
High-level design of the cGAN training scheme. For unconditioned GANs, the conditioning image is replaced with Gaussian noise; thumbnails exemplify the bone translation task outlined in Section 2.4.

With cGANs, data now emits paired samples {x,c}, which allows the final syntheses to be based on some limited information in the real dataset. This formulation is used for this study, where *x* is a TSE slice and *c* is the corresponding DESS slice, the same TSE slice with the inverse bone mask applied, or the corresponding DESS slice with the bone mask applied, as prescribed by each task. Conditioning the syntheses is essential to these tasks if each voxel in the synthesized slice and the real slice, which derive the anomaly map, is to correspond to the same location in the same patient. The high-level design of GANs is shown in Figure 5.

Under ideal training settings, *G* improves its generative capabilities so that $\hat{x}$ looks more real, and *D* simultaneously improves its discriminative capabilities such that $\hat{x}∼p_{g}$ can be separated from $x∼p_{data}$. In theory, this process can extend indefinitely until *G* and *D* reach a Nash equilibrium and $p_{g}=p_{data}$.

The specific implementation of cGAN in this study is inspired by Pix2Pix [27], known for its ability to generalize for different tasks without changing the network architecture. During generator training, L1 reconstruction loss is added to the loss from the value function in Equation (4):

(5) $$L_{L1}\left(G\right)=E_{x,z,c}||x-{G\left(z,c\right)||}_{1}$$

which is possible thanks to the existence of the ground truth target *x* in the paired training samples {x,c}. This loss encourages translations from c→x that are more accurate to that found in real dataset pairs. In particular, L1 distance between the real image and the reconstruction is used to minimize blurring, which would be commonplace if L2 distance were used.

Both generator and discriminator network architectures are based on U-Net [25], with convolution, batch normalization, and rectified linear unit (ReLU) sets for each layer and the signature skip connections. The discriminator is truncated at the bottleneck, then fitted with a binary classifier at its head.

#### 2.5.2. Conditional Diffusion Model

Denoising diffusion probabilistic models, or diffusion models, are another class of generative models recently popularized in 2020 by Ho et al. [28]. Diffusion models have been found to exhibit some strengths relative to GANs: lack of adversarial training process which leads to more stability during training; higher quality of synthesized samples; and the avoidance of mode collapse, in which the model fails to represent some high frequency modes in the training dataset. These advantages come at the cost of slower convergence during training as well as sampling during inference. This study tests these differences by comparing their performances in tasks described in Section 2.4.

Like with GANs, this study uses an extension to diffusion models; an image-to-image conditional diffusion model named Palette [29] is the primary inspiration for this section of the study. the noise model $\epsilon_{\theta }$ is modified to accept the conditional data *c* in the paired sample in {x,c}. Regardless of *t*, the original undisturbed *c* is sent to each step of the denoising process. L2 distance between the real and predicted noise is used, but additional reconstruction loss is not proposed, unlike with Pix2Pix.

Palette uses a U-Net architecture with increased depth, increased number of attention heads, varied attention patch sizes, BigGAN residual blocks [30], and rescaled residual connections as prescribed by Dhariwal and Nichol [31].

### 2.6. Anomaly Map Postprocessing

For each of the three tasks described in Section 2.4, the output of the model framework is the same: a voxel-wise anomaly map corresponding to a real TSE slice. Unlike a standard binary segmentation map for a specific lesion like BMEL, the anomaly map is a probabilistic topology of any regions that do not match the training data, which is made of individual slices of a knee that was predetermined not to have any BMEL. Therefore, some postprocessing is required to eliminate any false positives that may indeed be anomalous but are not BMEL, and to binarize the probabilities into a segmentation map.

First, bone masks are applied to the anomaly map such that any false positives outside of bone is eliminated. Then, to determine the binarization threshold value, the testing anomaly maps are split into three folds for a three-fold cross-validation. Here, expert annotations and the unsupervised method’s ability to match them are measured using DICE. For each validation fold, the other two folds are used to determine the optimal threshold value. A sweep of threshold values is used and the corresponding DICE scores for each of the annotators were computed and averaged. The threshold value with the highest DICE score is determined to be the optimal. The validation fold then uses the optimal threshold value to binarize its anomaly maps and compute its testing DICE score. This is repeated for each fold and the final reported DICE score is the arithmetic mean of the three testing DICE scores. This is done to prevent the model from overfitting to the combination of the provided expert annotations and threshold value.

## 3. Results

This section reports experimental results for the annotations and the unsupervised deep learning models. In evaluating the model performance, the annotations are treated as ground truths, even though we acknowledge and quantify their flaws in Section 3.1.

### 3.1. BMEL Annotation

**Figure 6:**
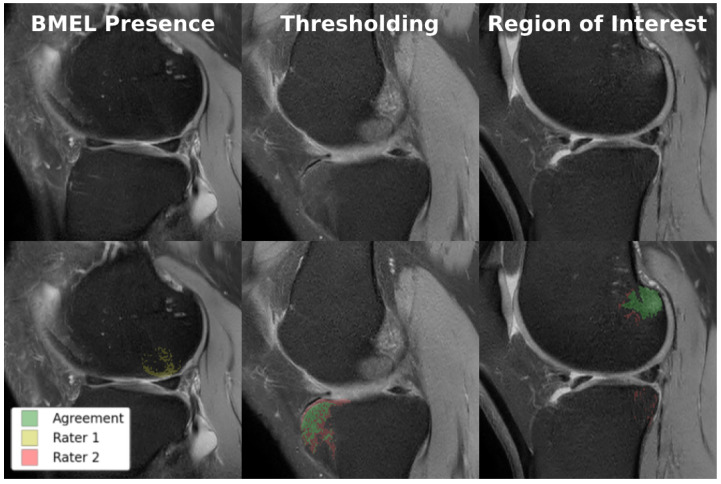
Examples of disagreement between raters in BMEL annotation. Top row: original TSE images; bottom row: TSE images overlaid with two annotations, where green represents voxels of agreement and yellow and red represents voxels of disagreement. Lannotators disagreed on whether BMEL was present in the slice (**left**); annotators disagreed on the thresholding value used to determine which voxels within the region-of-interest constituted BMEL (**center**); annotators disagreed on the extent of the region-of-interest (**right**).

Ten volumes of each from the MOON and COMET cohorts were held out from training and manually annotated for model performance and intra-/inter-rater reliability testing. All twenty volumes were annotated at least once by each of the two annotators, enabling inter-rater reliability measurements. The ten MOON volumes were annotated again by one of the annotators for intra-rater reliability measurements. Each of these two sets of annotations were compared against the other rater’s annotations to produce two DICE scores, which were averaged to produce a single metric. As shown in Figure 6, there were multiple sources of disagreement between the annotators, of which thresholding was the most significant. Table 3 quantifies the intra-/inter-rater reliability for BMEL annotations, including 2D DICE for all slices, which includes slices without any annotated BMEL, and 2D DICE for BMEL slices, which exclude slices without any annotated BMEL.

### 3.2. Deep Learning Segmentation

For each of the six model class and task combinations, the testing dataset was used to generate model predictions and compared against the raters’ annotations. 3D DICE scores were computed and averaged across the testing volumes for each rater, then averaged again across the raters. The final metrics are reported in Table 4.

**Figure 7:**
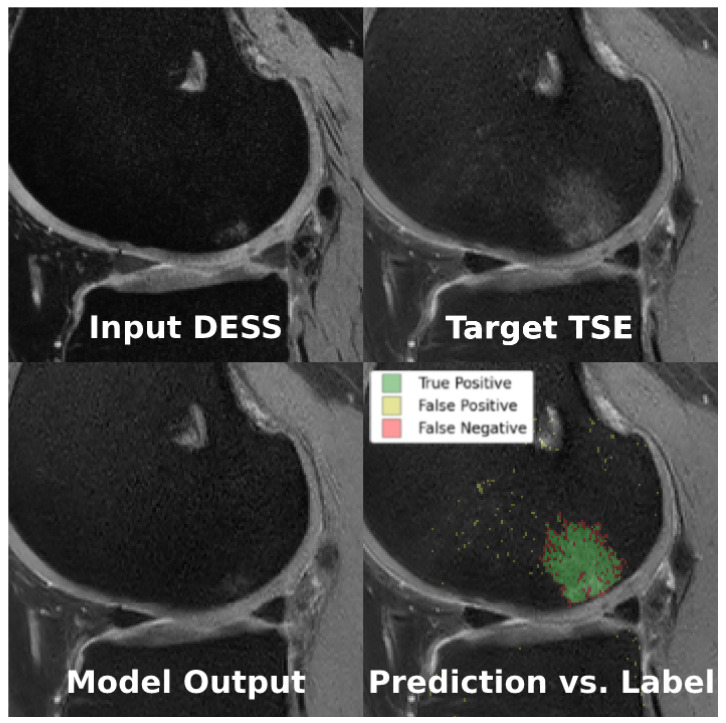
Example of the sequence translation task using cDIFF, where conditioning image is the real DESS image (**top left**), ground truth is the real TSE image (**top right**), and the model output is the fake TSE image (**bottom left**). When comparing the model prediction against the BMEL annotation (**bottom right**), green is true positive, yellow is false positive, and red is false negative.

The models performed similarly at their best, albeit on different tasks: bone inpainting and sequence translation for cGAN and cDIFF, respectively. Examples of the sequence translation task on cDIFF are shown in Figure 7. Another metric of note is the parameter count for each; cDIFF has nearly tenfold the number of parameters when compared to the cGAN. The best performing cDIFF model trained on the sequence translation task was configured as follows: batch size of 6; Adam optimizer with a fixed learning rate of $1\times 10^{-4}$ [32]; Kaiming model parameter initialization [33]; and a quadratic noise schedule with 1000 timesteps as described by Nichol et al. [34]. The best performaing cGAN model trained on the bone translation task was configured as follows: batch size of 36; Adam optimizer with a fixed learning rate of $5\times 10^{-4}$; model parameters initialized to zero; and PatchGAN discriminator with three convolutional layers. More details are given in Section 2.5.

## 4. Discussion

This study introduces an innovative unsupervised approach for the segmentation of BMELs in knee MRI, leveraging conditional diffusion models, conditional generative adversarial networks, and anomaly detection. This approach addresses significant challenges posed to traditional supervised segmentation methods in the context of lesion segmentation: poor intra- and inter-rater reliability among expert annotators; costly annotations; insufficient dataset sizes, and highly variable lesion shapes, sizes, and textures. This study proposes and successfully demonstrates the feasibility of an unsupervised framework that can segment BMELs without the aforementioned limitations. This study also introduces novel methods for conditioning the generative models, in the form of tasks. These tasks are able to exploit the availability of multiple sequences for each subject which has different contrasts of BMELs and the knowledge that BMELs present only in the bone marrow, with increased signal intensity.

In addition, this study also highlights the difficulties inherent in manual BMEL segmentation. Previous studies have evaluated intra-/inter-rater reliability for BMEL grading. To date, it is the first study that quantifies intra-/inter-rater reliability for quantitative BMEL segmentation and notes the low agreement among experts. This is concrete evidence that the problem of BMEL segmentation itself is difficult, whether a neural network is present in the solution or not. Future developments in BMEL identification, classification, and segmentation should be cognizant of this fact.

### 4.1. DICE Score Implementation

DICE score is the primary metric used to quantify intra-/inter-reliability, given in Equation (1). In practice, it is supplemented with an additional hyperparameter:

(6) $$\mathrm{DICE}=\frac{2\mathrm{TP}+\epsilon }{2\mathrm{TP}+\epsilon +\mathrm{FP}+\mathrm{FN}}$$

where ϵ, also called smoothing factor, is a small positive hyperparameter that stabilizes the score in cases where TP is very low. It also prevents divide-by-zero errors when TP=FP=FN=0. ϵ can have a significant impact on 2D DICE scores since many of the slices do not have any BMEL, as annotated by experts. In these cases, if ϵ=0 a divide-by-zero error occurs and the slice is ignored. On the other hand, if ϵ>0, then DICE=ϵ/ϵ=1, which skews the average score towards 1. 3D DICE does not suffer from this susceptibility to ϵ because each testing volume has at least some BMEL.

Focusing on 3D DICE, the low intra-rater agreement indicates that the segmentation of BMEL is an inherently difficult problem, even when controlling for the expert annotator. This may also be due to the inconsistency with which the annotation tools are used. The inter-rater agreement is even lower, indicating that experts disagree significantly on the exact boundaries of BMELs, even if they identify the same general region. These results are consistent with our hypothesis.

### 4.2. Generative Model Class and Size

Diffusion models are generally understood to have more output diversity and mode coverage relative to GANs [35]. In addition, more complex datasets do better with more model parameters whereas simpler datasets do better with fewer model parameters. Both of these factors may contribute to the best performing task for each model class: sequence translation for cDIFF and bone translation for cGAN. However, this reasoning is complicated by the fact that the aforementioned model class and parameter count comparisons are typically made based on general-purpose datasets such as ImageNet, which are much more diverse than the dataset used in this study. Without further investigations into the relative diversity and complexity of medical datasets, applying the generic reasoning to this study is questionable. Empirically, the nearly ten-fold larger parameter count of cDIFF did not have a positive impact on model performance relative to the cGAN.

### 4.3. Task Selection

During the experiments, we hypothesized that limiting the scope of the generative models to the least amount of information was the most effective setting for unsupervised BMEL segmentation. Since bone translation masks out the region surrounding the bone and precludes extraneous information, we expected both model classes to perform best on the bone translation task. In practice, the cGAN performed best on the bone inpainting task, while the cDIFF performed best on the sequence translation task. The non-bone regions, such as cartilage, tendon, and muscle, may contain some information that enabled cDIFF to better reproduce the bone region. Another possibility is that the excess number of parameters in cDIFF overfitted on simpler tasks, whereas it was a better fit for the sequence translation task. Future ablation studies may help narrow down these possibilities.

### 4.4. Related Works

A significant consideration in lesion segmentation studies is the diversity of the training and testing datasets, as well as the size of the lesions themselves. Muller et al. [36] noted that lesion segmentation in MRI is heavily impacted by the size of the lesion relative to the volume as a whole, and that DICE is a flawed metric in such cases. This is highlighted by the fact that existing BMEL segmentation and quantification studies rely on homogenous datasets, where individual BMEL lesions are larger [20] and/or require semi-automatic methods such as marking the center of each BMEL before passing it to the model [21]. Despite our lower DICE scores, our study is fully automatic, and most importantly, does not require any training data.

### 4.5. Limitations

The study acknowledges several limitations: low overall performance; reliance on expert annotations for model evaluation; the use of only two expert annotators; use of DICE as the only evaluation metric, and a lack of external validation/testing dataset. Our future plans include correcting these issues by collecting more data and observing the change in model training and performance, especially with the tracking of multiple segmentation metrics, such as intersection-over-union, and precision and recall.

## 5. Conclusions

This study is a positive step in the direction of knee osteoarthritis diagnosis, since it promotes the training of lesion segmentation models without the need for expert annotators. The methods proposed by this study can be extended to other anomaly detection tasks as well. Future research should focus on iterating on the model training configurations to confirm that they are performing optimally under the current design. This includes the expansion of the training dataset as well as testing on inhomogeneous datasets. Then, a hybrid, supervised and unsupervised approach could be explored, where the reliance on expert annotations can be controlled for during training. This may include the development of a new evaluation metric that is not dependent on expert annotations. Finally, future work should incorporate the measures of ultimate interest, that of knee osteoarthritis detection, classification, treatment, and cure. By expanding the scope of the study, we hope to reach the ultimate goal of improving patient outcomes through quantitative medical imaging.

## Figures and Tables

**Table 1:** The demographics of the subjects used in model training and testing. Subjects from each of the MOON and COMET trials were used for both training and testing.

Study	Count	Age (Mean)	Race (%)	Sex (% Female)
MOON	n=166	30.5	White—91	53
			American Indian—2	
			Black—3	
			Asian—2	
			Other—2	
COMET	*n* = 11	53.6	White—55	36
			Other—18	
			Unknown—27	

**Table 2:** Acquisition protocol for sagittal TSE and DESS sequences used in this study.

	MOON		COMET	
**Sequence**	Sagittal TSE	Sagittal DESS	Sagittal TSE	Sagittal DESS
**Time (m:s)**	2:18	5:56	2:43	5:56
**TR/TE (ms)**	2490/18	17.55/6.02	2930/17	17.55/6.02
**Matrix**	320 × 256 × 33	384 × 307 × 160	320 × 256 × 35	384 × 307 × 160
**Slice Thickness (mm)**	3	0.7	3	0.7

**Table 3:** 3D Dice Similarity Coefficient (DICE) averaged across volumes; 2D DICE is averaged across slices.

	3D DICE	2D DICE	2D DICE
		**(All Slices)**	**(BMEL Slices)**
Intra-Rater	0.7123	0.9500	0.6155
Inter-Rater	0.6423	0.8763	0.4725

**Table 4:** 3D Dice Similarity Coefficient (DICE) averaged across volumes and raters for each model class and task combinations.

	Parameters (millions)	DICE per Task		
		**Sequence Translation**	**Bone Inpainting**	**Bone Translation**
cGAN	7.312	0.1598	0.2299	0.1409
cDIFF	62.638	0.2244	0.0491	0.1045

## Data Availability

The datasets presented in this article are not readily available because of privacy and ethical concerns. Deidentified data will be made available upon request following the completion of the study.
